# Identifying Attrition Phases in Survey Data: Applicability and Assessment Study

**DOI:** 10.2196/12811

**Published:** 2019-08-23

**Authors:** Camille J Hochheimer, Roy T Sabo, Robert A Perera, Nitai Mukhopadhyay, Alex H Krist

**Affiliations:** 1 Department of Biostatistics Virginia Commonwealth University Richmond, VA United States; 2 Department of Family Medicine and Population Health Virginia Commonwealth University Richmond, VA United States

**Keywords:** patient dropouts, surveys and questionnaires, survival analyses, statistical models

## Abstract

**Background:**

Although Web-based questionnaires are an efficient, increasingly popular mode of data collection, their utility is often challenged by high participant dropout. Researchers can gain insight into potential causes of high participant dropout by analyzing the dropout patterns.

**Objective:**

This study proposed the application of and assessed the use of user-specified and existing hypothesis testing methods in a novel setting—survey dropout data—to identify phases of higher or lower survey dropout.

**Methods:**

First, we proposed the application of user-specified thresholds to identify abrupt differences in the dropout rate. Second, we proposed the application of 2 existing hypothesis testing methods to detect significant differences in participant dropout. We assessed these methods through a simulation study and through application to a case study, featuring a questionnaire addressing decision-making surrounding cancer screening.

**Results:**

The user-specified method set to a low threshold performed best at accurately detecting phases of high attrition in both the simulation study and test case application, although all proposed methods were too sensitive.

**Conclusions:**

The user-specified method set to a low threshold correctly identified the attrition phases. Hypothesis testing methods, although sensitive at times, were unable to accurately identify the attrition phases. These results strengthen the case for further development of and research surrounding the science of attrition.

## Introduction

### Background

Web-based surveys and questionnaires are an increasingly popular mode of data collection because of factors such as the ease of delivery, cost-effectiveness, automated data management, and ability to reach a large pool of respondents. Compared with participants of paper-based surveys, Web-based survey participants have greater freedom to drop out at any point, especially when they feel that the questionnaire is no longer relevant to them. This results in dropout attrition, where a participant starts but does not complete the survey [1,2]. Consequently, the rate of dropout attrition is often much higher in Web-based surveys than their paper-based counterparts [1,3]. Dropout attrition is different from nonresponse attrition, for which the research is widely established, where participants are solicited but choose not to participate in a survey [1,2,4,5]. For the purposes of this paper, dropout and attrition will both refer to dropout attrition.

Identifying distinct phases of dropout also presents a missing data problem. When dropout is associated with topics addressed by the survey itself, it may be considered either missing at random (MAR) or missing not at random (MNAR) [6]. Ignoring data MAR or MNAR may result in a biased inference when analyzing data, where statistical significance changes depending on whether or not the missing data are addressed [7]. Thus, when attrition phases are present, a researcher may consider using different methods to evaluate the results that account for data MAR or MNAR.

In Eysenbach’s original call for a *science of attrition*, he introduced the idea that survey attrition occurs in distinct phases: the *curiosity plateau* at the beginning of a survey where the participation rate remains high while respondents gauge their interest in the survey, the *attrition phase* where participants exit at a higher rate, and the *stable use phase* where most remaining participants are likely to complete the survey [1]. We previously responded to Eysenbach’s call with a 3-step process for investigating where and why attrition occurs by first visualizing dropout trends, then confirming statistically significant dropout, and finally exploring factors associated with attrition [8].

Although our previous study suggests that visualization of dropout patterns (particularly plotting the number of dropouts at each question) provides a reasonable estimation of attrition phases, a natural next step would be to directly estimate Eysenbach’s phases of attrition using statistical methods. If clear attrition patterns are identified, this suggests that the dropout is because of the content of the questionnaire itself (research suggests that it is the survey content rather than the survey length that drives participant dropout [9-11]) and thus, any further analyses of the survey data should account for both the missing data and the mechanism by which their missingness differs. Identifying the attrition phases not only highlights questions where dropout is high but also classifies the entire series of questions with similar dropout rates; doing so in an empirical manner also reduces researcher reliance on subjective classifications.

### Objectives

This paper aimed to describe the application of clinical thresholds and existing statistical methods to the task of identifying Eysenbach’s phases of attrition. In the Methods section, we first propose an approach searching for clinically or practically meaningful attrition through user-specified thresholds. We also propose the novel application of 2 existing statistical techniques to identify the phases of attrition in survey data. In the Results section, we conduct a simulation study and apply all 3 approaches to the results of a Web-based survey about cancer screening to demonstrate the performance of these methods for identifying phase transitions. Finally, in the Discussion section, we suggest future directions for this research.

## Methods

### Methods for Identifying Eysenbach Phases of Attrition

We have proposed an approach motivated by establishing meaningful dropout standards and 2 additional approaches motivated by statistically significant attrition. Statistical significance is not always indicative of a meaningful difference, especially when the sample size is large, as is often the case with Web-based surveys. Thus, these serve as complementary methods to identify clear inflection points of the dropout rate.

#### User-Specified Attrition Thresholds

For the first proposed method, the researcher specifies the amount of dropout that they consider to be clinically or practically meaningful, allowing them to choose the sensitivity of this method. Specifically, they define thresholds for the start and end of the dropout phase of attrition. The proportion of dropout for each survey question is then compared with both the thresholds. When applying this method, the first question for which dropout exceeds the start threshold was interpreted as the beginning of the dropout phase and the last time that dropout exceeds the end threshold was interpreted as the end of the dropout phase. Note that different numerical values could be used for the start and end thresholds.

#### Hypothesis Testing Methods

For the following 2 hypothesis testing methods, we fit the model to the entire survey and then apply successive differences contracts to test pairwise differences in the dropout rates between adjacent questions; note that here the outcome is a binary dropout indicator and not the original survey response. The null hypothesis that dropout rates do not change between questions was rejected in favor of the alternative hypothesis that the rates were different between questions when the corresponding *P* value was lower than an appropriately adjusted significance level. We assumed dropout monotonically increased throughout the survey. The first and last instances of statistically significant differences in the dropout rate between questions were interpreted as the start and end of the dropout phase of attrition. *P* values were adjusted for multiple comparisons using Benjamini and Hochberg’s false discovery rate correction, with adjusted *P* values evaluated at the 5% level [12].

##### Generalized Linear Mixed Model

Survey questions are individual, discrete units that are dependent within survey participants. In other words, once a participant drops out of a survey, they cannot answer any further questions. A generalized linear mixed model (GLMM) using the logit link can account for these features while allowing researchers to compare differences in the proportion of respondents between sequential questions. In this model, the binary outcome was whether or not a participant had dropped out of the survey by that question. Question number was included as a time-varying covariate with the number of levels equal to the number of questions and an indicator function identifying the question of interest. A subject-level random effect was included to account for within-subject dependence between response rates, accounting for the fact that a participant cannot reenter the survey once they have dropped out.

##### Discrete Time Survival Analysis

Discrete time survival analysis (DTSA) is another appropriate method for analyzing survey attrition because questions occur in a discrete order and respondents can only dropout at these distinct points. We propose treating dropout as a time-to-event outcome, meaning we counted how many questions were answered before the respondent dropped out and used this as a time-to-event outcome. In the survival setting, we inherently assume participants are followed over time, allowing us to eliminate the subject-level random effect. The multivariate, dependent set of outcomes was replaced with a minimally sufficient outcome, meaning no information was lost in this simplified model.

The outcome of this model was whether or not the participant experienced the event of survey dropout. The baseline hazard function was a step function with a dummy variable for each question at which the participant could drop out (which is the last question if they completed the survey); thus, we tested for changes in the hazard of dropping out of the survey [13].

### Simulation Study

To compare the performance of these methods in detecting attrition phases, we simulated a variety of dropout patterns. For each pattern, we simulated 10,000 datasets, each with 200 simulated participants answering 20 questions. Respondents had a random chance of dropping out at any point in the survey, including the first question, and a participant could not reenter once they had dropped out.

**Figure 1:**
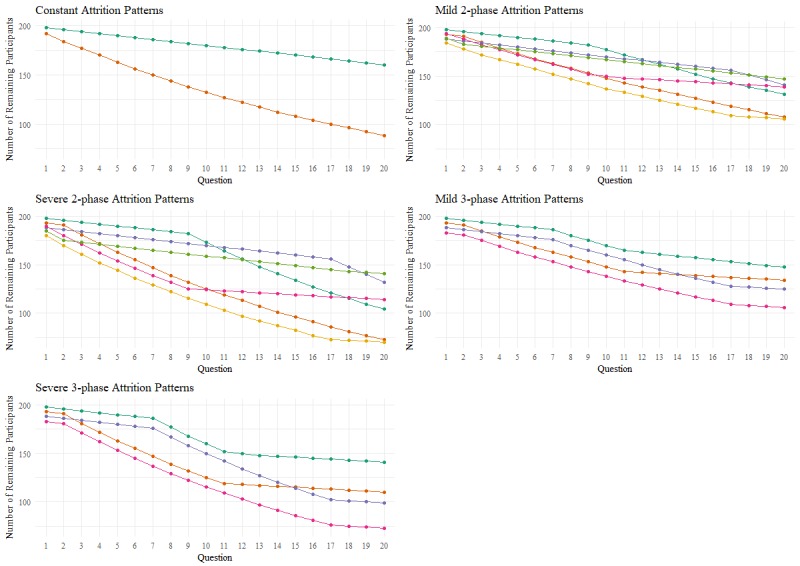
Simulated attrition patterns.

Simulated dropout patterns corresponded to constant, 2-phase, and 3-phase attrition. Constant attrition could be either the stable use or dropout phase of attrition throughout the survey (see the top-left panel of Figure 1). Two-phase attrition either began with the stable use phase and then transitioned into the dropout phase for the remainder of the survey or began with the dropout phase with a transition at some point during the survey into the stable use phase (see the top-right and middle-left panels of Figure 1). Three-phase attrition followed Eysenbach’s proposed pattern in that there were stable use phases at the start and end of the survey with a dropout phase in the middle (see the middle-right and bottom panel of Figure 1). We tested both mild and severe attrition rates for dropout phases, where severe attrition rates demonstrated more pronounced differences in dropout rates between phases, to determine the sensitivity of these methods. The location of the phase transition was varied to see whether these methods better identified phase transitions that occurred near the start, middle, or end of the survey.

The overall null hypothesis being tested was that there are no phases of attrition, represented by the constant attrition patterns in the top-left panel of Figure 1. When the dropout rate was mild, we expected our proposed methods would not detect practically meaningful or statistically significant attrition at any point in the survey. When this rate was severe, we expected the methods to detect the first and last instances of significant attrition at the start and end of the survey, again suggesting constant attrition throughout. In other words, if the dropout rate was constant but significant, we interpreted this result as the dropout phase occurring throughout the survey.

When implementing the user-specified method, we assessed user-specified thresholds of 3%, 5%, and 8%. Although these are arbitrary thresholds, we found that using a sensitive 3% threshold was the only threshold that was able to distinguish attrition patterns in both the simulation study and the test case application (discussed below); thus, only the results of the 3% threshold are presented.

We determined how well these methods achieved the goal of detecting phases of attrition by calculating and comparing the type I error and sensitivity of each method. Here, type I error was defined as when at least 2 phases of attrition were detected when the underlying attrition pattern was constant (ie, phases of attrition were detected when they did not exist). Sensitivity was defined as finding the correct number of phase transitions when they did exist. Ideally, these methods would achieve a type I error of 5% and higher sensitivity.

We also visually inspected how often each question was chosen as the start or end of the attrition phase by plotting these distributions with histograms. The histograms for the user-specified method highlight the first and last question at which the amount of dropout at a question surpassed 3%; histograms for the GLMM and DTSA show the first and last comparisons between questions where there was a significant adjusted *P* value.

### Test Case

Our test case data were from a Web-based survey entitled the informed decision-making (IDM) module. The 17-question survey explored how patients approach decisions regarding screening for breast, colorectal, and prostate cancer. Here we focus exclusively on the results for colorectal cancer, where there were 1249 participants. Questions addressed the awareness of screening eligibility, screening options, primary concerns about cancer screening, and planned next steps [14]. This survey was designed by the Virginia Commonwealth University Department of Family Medicine and Population Health research team and administered from January to August, 2014, in 12 primary care practices throughout northern Virginia through the interactive Web-based patient portal, *MyPreventiveCare* [15-18]. More specific details regarding the survey, including screenshots of the questionnaire itself, can be found in the studies by Hochheimer et al and Woolf et al [8,19].

All simulations and analyses were conducted using the R statistical software version 3.5.0 [20]. When applying the user-specified method, we used a threshold of 3%, 5%, and 8%, although only the results of the 3% threshold are discussed because of the poor performance of the 5% and 8% thresholds. The *survey* package was used to apply DTSA with questions treated as categorical factors of equal weight [21]. A successive differences contrast was applied to the results of both hypothesis testing models using the *multcomp* package to test each pairwise difference in the proportion of participants remaining in the survey and the hazard of dropping out between questions for the GLMM and DTSA, respectively [22]. All figures in this paper were created using the *ggplot2* package [23].

## Results

### Simulation Study Results

**Figure 2:**
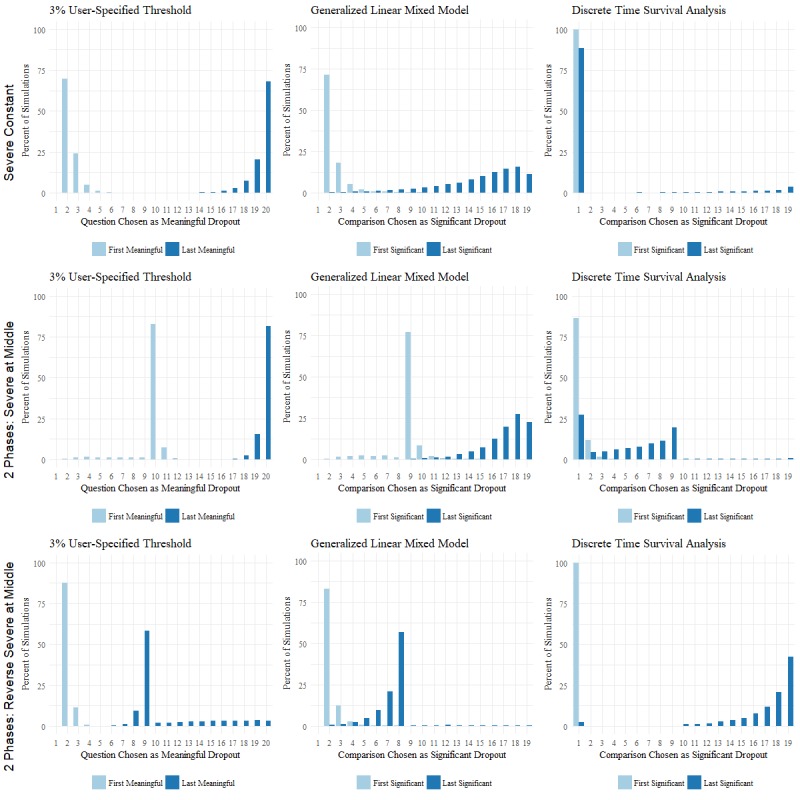
Selected bar charts displaying the percent of simulations where each question was chosen as the first and last instance of meaningful/significant attrition.

The resulting type I error and sensitivity of each method for each simulated attrition pattern can be found in Table 1. A selection of the histograms displaying the distribution of questions chosen as the start and end of the attrition phase is displayed in Figure 2. In the case of severe constant attrition, we expected to see the first instance of meaningful attrition at question 1 for the user-specified method and the first instance of significant attrition between questions 1 and 2 for the GLMM and DTSA. Then, we hoped to see this attrition phase last throughout the survey, with the last instance of meaningful attrition found at the final question and significant attrition found between the last two questions.

#### User-Specified Attrition Thresholds

The 3% user-specified threshold had high type I error but also high sensitivity to detect attrition phases, especially in cases of a severe dropout phase and 3 phases of attrition. This threshold achieved higher sensitivity than the other 2 methods when the simulated dropout pattern for 2 phases began with the dropout phase and ended with the stable use phase.

The user-specified method failed to identify the start of the severe constant attrition phase immediately at question 1 (see left column of Figure 2). In the majority of simulations, this method correctly detected severe attrition phases simulated to last from questions 1 to 10 and from questions 10 to 20.

#### Generalized Linear Mixed Model

The GLMM had a conservative type I error rate in the case of constant mild attrition and was unable to control type I error in the case of constant severe attrition. This method demonstrated low sensitivity to detect 2 phases of attrition in general and 3 phases with a mild dropout phase. The GLMM had higher sensitivity to detect all 3 of Eysenbach’s attrition phases in simulation patterns with a severe dropout phase.

The histograms in the middle column of Figure 2 reveal that the GLMM failed to identify the start of the attrition phase immediately at question 1 when the simulated pattern was that of severe constant attrition. These plots also show that when a severe attrition phase was simulated to begin in the middle of the survey and continue until the end of the survey, the GLMM most often detected the start of the attrition phase correctly at question 10 but detected the end of the attrition phase before the end of the survey (resulting in the low sensitivity seen in Table 1). When a severe attrition phase was simulated to begin at the start of the survey and last until the middle of the survey, the GLMM failed to identify an immediate start to the attrition phase but correctly distinguished the phase transition at question 10.

**Table 1:** Simulation study results.

Metric, simulation type, and attrition severity			Location of phase transition	3% user-specified threshold	GLMM^a^	DTSA^b^
**Type I error**						
	**Constant**					
		Mild	—^c^	0.19	0.01	0.74
		Severe	—^c^	0.53	0.98	0.96
**Sensitivity**						
	**Two phases**					
		Mild	Middle	0.47	0.22	0.90
		Mild	Start	0.48	0.17	0.94
		Mild	End	0.72	0.13	0.87
		Mild reverse	Middle	0.44	0.20	0.64
		Mild reverse	Start	0.51	0.01	0.69
		Mild reverse	End	0.41	0.14	0.58
		Severe	Middle	0.82	0.24	0.91
		Severe	Start	0.79	0.20	0.97
		Severe	End	0.90	0.56	0.88
		Severe reverse	Middle	0.88	0.02	0.58
		Severe reverse	Start	0.90	0.01	0.69
		Severe reverse	End	0.88	0.00	0.50
	**Three phases**					
		Mild	Middle	0.67	0.12	0.08
		Mild	1 start	0.96	0.32	0.07
		Mild	1 end	0.96	0.35	0.05
		Mild	Ends	0.96	0.51	0.03
		Severe	Middle	0.98	0.84	0.08
		Severe	1 start	0.96	0.99	0.07
		Severe	1 end	0.96	0.99	0.06
		Severe	Ends	0.94	0.99	0.04

^a^GLMM: generalized linear mixed model.

^b^DTSA: discrete time survival analysis.

^c^Not applicable in the case of constant attrition.

#### Discrete Time Survival Analysis

DTSA did not control type I error and had low sensitivity to detect 3 attrition phases. For simulated patterns with 2 phases, DTSA demonstrated high sensitivity to detect both the phases. DTSA achieved higher sensitivity than the user-specified and GLMM approaches for simulated patterns with a mild change in attrition between phases as well as when the simulated dropout pattern survey began with the stable use phase and transitioned to a severe dropout phase (Table 1). Overall, we observed higher sensitivity for DTSA when the simulated dropout pattern began with the stable use phase and then transitioned to the dropout phase compared with patterns beginning with the dropout phase and ending with the stable use phase. DTSA also consistently had higher sensitivity when the simulated phase transition occurred toward the start of the survey.

Histograms displaying the accuracy of DTSA can be found in the right column of Figure 2. The comparison between questions 1 and 2 was correctly recognized as the first instance of significant attrition but also incorrectly chosen as the last instance when the dropout phase was simulated to last throughout the survey. Although this method detected the correct number of phases in the majority of simulations with 2 phases, it did not choose the correct questions as the start and end of the attrition phase. Specifically, DTSA was unable to detect an abrupt change in dropout rate in the middle of the survey. The histograms suggest a dropout phase at the beginning of the survey when the underlying simulated pattern had a dropout phase in the second half of the survey and suggest constant attrition when the simulated pattern had a dropout phase in the first half of the survey.

### Results of the Informed Decision-Making Module Application

First, we inspected a plot of the number of dropouts at each question of the IDM module for colorectal cancer as suggested in the study by Hochheimer et al [8]. We observed high attrition from questions 3 to 5 with another spike at question 9. We hypothesized that our proposed methods would detect the dropout phase to last from questions 3 to 9 and the 3% user-specified threshold was able to detect exactly that. The GLMM was unable to detect any significant changes in the dropout rate throughout the survey. Finally, DTSA was only able to detect the start of the dropout phase. The results suggested a significant increase in the hazard of dropping out between questions 2 and 3 but also that the dropout phase lasted until the end of the survey. This is inconsistent with the observed dropout pattern, where we saw visual proof of the stable use phase from questions 10 to 17.

## Discussion

### Principal Findings

In our simulation study, none of the 3 proposed methods consistently detected the correct number of phases while controlling type I error. The 3% user-specified threshold had a high type I error rate but also accurately detected the phases of attrition and had moderate to high sensitivity for all simulated scenarios. Although high sensitivity estimates of DTSA in the case of 2 attrition phases appeared promising, histograms revealed that this method consistently identified the wrong questions as the start and end of the dropout phase. This explains the low sensitivity of DTSA to detect all 3 phases of attrition. DTSA was extremely sensitive, finding a significant difference between the first 2 questions even when the simulated dropout rate was very small (eg, 0.001).

We did not see any distinct patterns in sensitivity when the phase transitions occurred toward the start or end of the survey compared with the middle of the survey. Sensitivity was often higher for the hypothesis testing methods when there was a sudden increase in attrition than when there was a sudden decrease in attrition. This suggests that these methods do not consistently detect a phase transition when dropout starts off at a high rate and then levels off at a certain point in the survey. This issue persisted even when the change was more pronounced, as it was in the severe cases. Although this should limit the ability of the GLMM and DTSA to detect 3 phases of attrition, we actually observed increased sensitivity for the GLMM when 3 phases were present.

We also investigated user-specified thresholds of 5% and 8%. The 5% threshold was unable to control type I error, with 0% type I error in the mild constant case and 94% type I error in the severe constant case. The 8% threshold had low sensitivity (often 0%) to detect any phase transition. Those of the 3% threshold had the best outcomes, both in type I error and sensitivity, suggesting that lower user-specified thresholds perform better at identifying the attrition phases.

When applied to our test case data, the 3% user-specified threshold was the only method able to detect the dropout phase from questions 3 through 9. The GLMM was not sensitive enough to detect a distinct dropout phase and DTSA was able to detect the abrupt increase in the hazard of dropping out between questions 2 and 3 but not the abrupt decrease between questions 9 and 10.

### Limitations

One important difference between this study and our previous study is that participants were able to drop out at the first question. Previously, having 100% compliance in the first question limited the number of questions to 10 when applying the GLMM because of convergence issues. By assuming simulated participants could drop out at question 1, we were able to apply the GLMM to the entire survey and compare this method with the other 2 discussed here. The methods discussed in this paper apply specifically to dropout attrition and do not address nonresponse or longitudinal attrition (see the discussion in the study by Hochheimer et al [8]). By assuming dropout monotonically increases throughout the survey, these methods do not account for the functionality to skip questions. Finally, although the greatest region of dropout in the IDM module was from questions 3 to 5, it could be argued that there were 2 dropout phases, with the second occurring around question 9.

### Future Studies

Instead of searching for the instance where dropout exceeds a set threshold, the user-specified method could also search for when the difference in the dropout rate between 2 sequential questions exceeds the threshold. Pooled logistic regression is yet another strategy to identify the dropout patterns without modeling between question dependence, with an interpretation closer to that of DTSA [24,25]. Although we are interested in the exact question or questions at which the attrition rate changes, our results suggest that researchers should not search for these inflection points question by question. Future directions will include a search for a method to model overall patterns that in turn reveal significant changes in dropout or hazard rate at particular questions, potentially through change-point modeling.

### Conclusions

Our research suggests that when applying practical thresholds and existing statistical methods to the task of identifying Eysenbach’s phases of attrition, sensitive user-specified thresholds correctly identify dropout phases at the cost of high type I error, whereas hypothesis testing methods are unable to correctly identify these phases. As we continue to advance the science of attrition, these results strengthen the case for developing new methods to identify attrition phases.

## Abbreviations

DTSA: discrete time survival analysis
GLMM: generalized linear mixed model
IDM: informed decision-making
MAR: missing at random
MNAR: missing not at random
